# Optimizing the lighting environment in college teaching buildings in the Changsha-Zhuzhou-Xiangtan region based on hybrid lighting patterns

**DOI:** 10.1371/journal.pone.0359574

**Published:** 2026-10-01

**Authors:** Leyan Zhang, Bi’nan Zuo, Xi Jin, Canlong Liu, Yufeng He

**Affiliations:** 1 Department of Architecture, Hunan University of Science and Technology, School of Architecture and Design, Xiangtan, China; 2 Department of Architecture, Hunan University of Technology, School of Civil and Environmental Engineering, Zhuzhou, China; Central Queensland University, AUSTRALIA

## Abstract

With the rapid expansion of higher education campuses in China, teaching buildings have become major contributors to lighting-related energy consumption. Most university teaching buildings adopt interior corridor layouts, in which classrooms rely primarily on single-sided daylighting, often resulting in insufficient daylight availability, particularly in north-facing spaces. Improving the lighting performance of interior corridor–type teaching buildings is therefore critical for enhancing energy efficiency and indoor environmental quality. In this study, an evaluation framework for indoor lighting environments is established, and the Chongye Teaching Building at Hunan University of Technology is selected as a representative case. Through a combination of on-site measurements and daylighting simulations, the daylighting characteristics of interior corridor–type teaching buildings in the Changsha–Zhuzhou–Xiangtan (Chang–Zhu–Tan) region are systematically analyzed. Four hybrid lighting patterns integrating natural and artificial lighting are proposed, including High-Density Layout (HDL), Medium-Density Layout (MDL), Low-Density Layout (LDL), and Targeted Layout (TL). The results indicate that all four patterns significantly enhanced daylight utilization across multiple metrics—including illuminance, spatial uniformity, daylight factor, spatial daylight autonomy, and target illuminance duration—thereby reducing dependence on electric lighting during daytime classroom operation. Through this proof of concept, the present study provides guidance and references for optimizing the luminous environment in interior-corridor classrooms under comparable architectural and daylight climate conditions.

## 1. Introduction

With the continuous expansion of higher education institutions in China, teaching buildings have become major consumers of lighting energy. Between 2012 and 2022, lighting energy consumption in educational buildings accounted for more than 50% of total electricity use, representing approximately 20% of overall building energy consumption [1]. Consequently, reducing lighting energy demand and promoting the development of energy-efficient and sustainable university buildings have become central objectives in contemporary campus planning and architectural design.

The Changsha–Zhuzhou–Xiangtan(Chang-Zhu-Tan) region is characterized by a high concentration of higher education institutions, with nearly 30 universities and colleges. Approximately 80% of the teaching buildings in this region feature an internal corridor layout, where corridors are located at the center of the building and teaching spaces are arranged on either side. The interconnection of rooms through these corridors forms a spatial configuration commonly applied in educational, office, and healthcare buildings. Despite its widespread use, this corridor-style layout presents several architectural and environmental drawbacks, including excessive glare from direct sunlight, uneven daylight distribution from side windows, increased dependence on artificial lighting, compromised indoor air quality, and elevated energy consumption. These issues pose significant challenges to the lighting environment of internal corridors, particularly in terms of effectively utilizing natural daylight, guiding and controlling light penetration, and enhancing overall lighting quality in an energy-efficient manner.

In terms of design technology, Lü Jiaqi [2], Chen Yidan [3], and Stanislav Darula [4] focused on the control and guidance of incident daylight in buildings. They proposed hybrid light-collecting approaches, daylighting system designs, adaptive building façades, and beam-focusing light-guiding systems for architectural applications. These patterns were evaluated through simulation-based analyses of illuminance levels, reflected light indices, glare, and daylight uniformity using real-world case studies. De Luca [5] demonstrated that the multi-objective optimization of static shading devices—considering glare control, daylight availability, visual connection, and energy performance—can reduce visual discomfort by 89.8% and overall energy consumption by 29.1%. Bellazzi [6] compared artificial skylights, natural daylight, and LED lighting systems, highlighting the potential of artificial skylights to reproduce daylight-like effects, while noting that related design metrics require further refinement. Mangkuto [7] and Kontadakis [8] employed simulation tools such as Rhinoceros, EnergyPlus, and Radiance to analyze daylighting performance and energy consumption, emphasizing the importance of multi-scenario optimization in architectural design. Stanislav Darula [9] conducted synchronized indoor–outdoor illuminance measurements on two tubular light guide systems with identical geometries but different inner surface coatings. The study systematically analyzed the effects of geometric parameters and reflectance combinations under standard CIE clear-sky and overcast-sky conditions, producing efficiency charts that offer practical guidance for daylighting system design. Guo Xuran [10] provided a comprehensive review of the design principles, manufacturing processes, evaluation methods, and performance characteristics of microprism materials used in complex fenestration systems. Zhao Xiaolong [11] proposed a novel light-shaping frame system incorporating folded reflectors, achieving dynamic and precise daylight control through a modular mechanical structure and coordinated control algorithms. Zhongcheng Duan [12] combined surrogate modeling with multi-objective optimization to integrate building performance simulation, machine-learning models, and intelligent algorithms, enabling coordinated optimization of energy use, daylighting, and thermal comfort.

In terms of design patterns, Hajji Apif M [13]. assessed daylighting performance in school classrooms using VDV software, identified insufficient illuminance levels, and recommended optimizing window-to-wall ratios to improve visual comfort. Overen Ochuko Kelvin [14] and Jayakumari Sujan Dev Sureshkumar [15] proposed the integration of photovoltaic systems with daylighting patterns as an emerging approach to enhance both energy efficiency and indoor lighting performance.

From the perspective of design methodology, Huang Bochao and Su Xiaoming [16], Guo Weihong and Liang Sheng [17], among others, investigated factors influencing natural daylighting from three dimensions: educational building spatial design, daylighting components, and overall building configuration. Through empirical optimization, they proposed daylighting design processes applicable to teaching buildings in local universities.

With respect to evaluation metrics, Wang Yiheng [18], Yang Liuliu and Li Zhenquan [19], and Lu Zhen and Huang Xianfeng [20] identified daylight autonomy (DA), useful daylight illuminance (UDI), annual sunlight exposure, and daylight glare probability as key indicators of visual comfort in architectural lighting. These studies further examined the impacts of design variables such as building depth, bay width, orientation, sill height, and exterior window dimensions.

The optimization of lighting environments in university teaching buildings has evolved into an integrated technical framework, progressing from passive design patterns to active control systems and further toward multi-objective optimization and real-time simulation. Current research primarily aims to achieve a balanced integration of visual comfort, non-visual health benefits, and building energy performance through the application of dynamic evaluation metrics and multi-objective optimization algorithms. However, regional specificity remains insufficiently addressed. Although previous studies have examined multiple climatic zones in China and conducted case-based analyses in cities such as Hohhot, Guangzhou, and Tianjin, investigations focusing on the Chang–Zhu–Tan region are still limited. The distinctive climatic conditions of this region, together with its substantial stock of existing buildings and high-density educational activities, pose both unique challenges and significant opportunities for the optimization of lighting environments in university teaching buildings.

According to China’s Standard for Daylighting Design of Buildings (GB 50033–2013), the Chang-Zhu-Tan region is located within Daylighting Climate Zone Ⅳ. Compared to other daylighting zones, this region receives significantly less direct solar radiation, resulting in relatively limited daylight resources. This study aims to address the problems of inadequate indoor lighting quality and high lighting energy consumption in interior corridor–type teaching buildings at universities in the Chang–Zhu–Tan region, which are mainly attributable to single-sided daylighting. The Chongye Teaching Building at Hunan University of Technology was selected as the case study to examine the daylighting characteristics of interior corridor–type teaching buildings, delineate effective daylighting zones and supplementary lighting zones, and develop four hybrid daylighting patterns incorporating natural light-guiding devices [21]. A combined approach integrating on-site measurements and numerical simulations was then adopted to evaluate the performance of the four modes in terms of illuminance, daylight uniformity, daylight factor, compliant area ratio, and compliant duration, and to identify the hybrid daylighting optimization pattern most suitable for university classrooms in the Chang–Zhu–Tan region. Finally, this study provides a reference for the optimization of lighting environments in interior corridor–type teaching buildings in the Chang–Zhu–Tan region and other regions with similar characteristics.

The remainder of this paper is organized as follows: The Materials and methods section describes the research methodology, including field measurement procedures and simulation parameters. The Results section reports and analyzes the measured and simulated results, highlighting existing lighting deficiencies. The Discussion section introduces the four hybrid daylighting patterns and compares their performance. Finally, the Conclusions section summarizes the main conclusions, discusses the limitations of the study, and suggests directions for future research.

## 2. Materials and methods

It should be noted that this paper presents a pilot study, which focuses on evaluating the physical performance of the proposed composite daylighting device and establishing the structural modes, thereby laying a foundational baseline for the deployment of intelligent algorithms in future research. To ensure clear reproducibility and systemic clarity, the holistic scientific workflow of this research is formalized into a coupled method-and-discussion framework (Fig 1).

**Fig 1:**
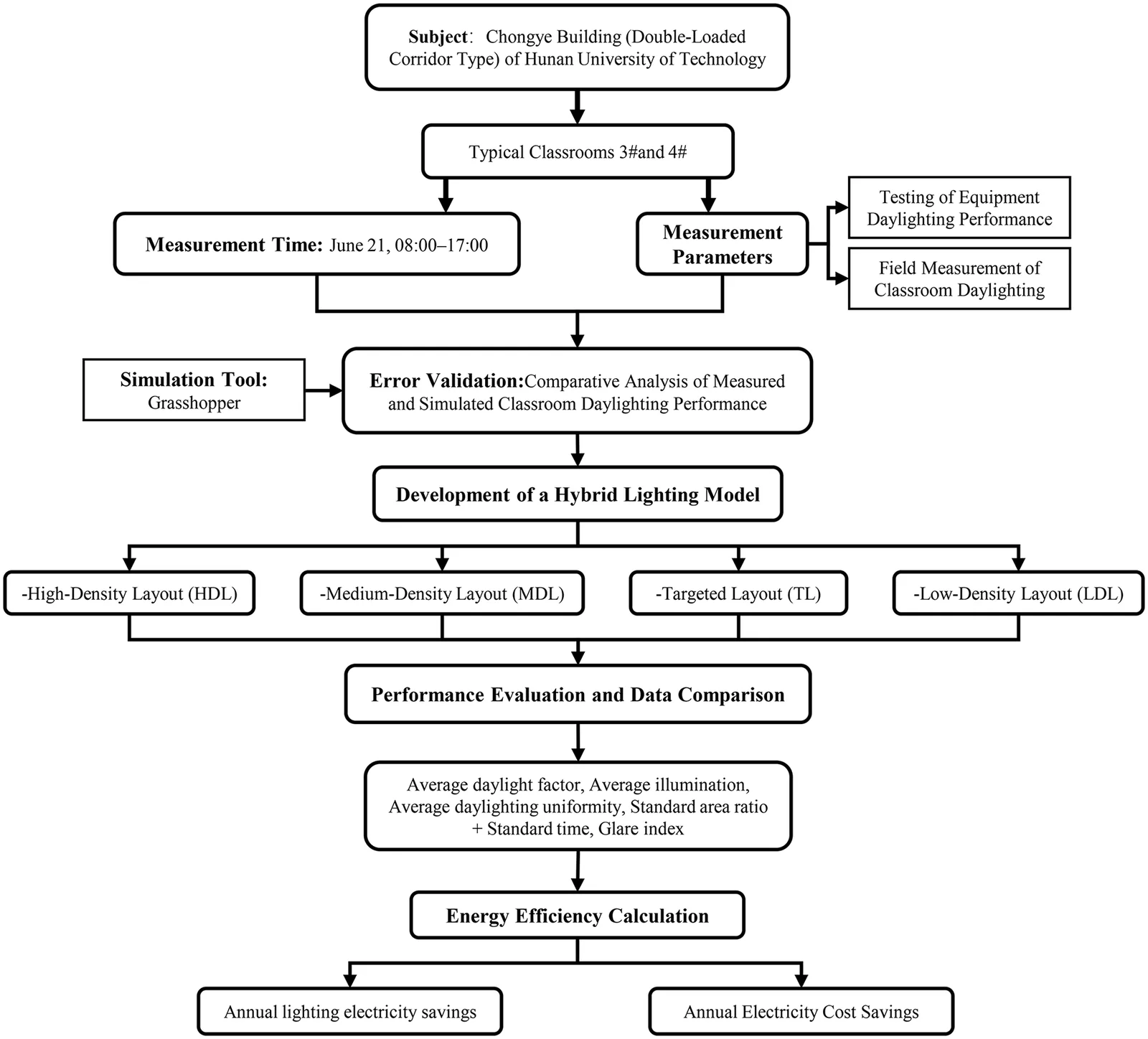
Technical roadmap of the research.

### 2.1. Environmental conditions

According to GB 50033–2013, the Chang-Zhu-Tan region is classified as a photometric climate zone IV (Table 1), with an annual average global illuminance (Eq) ranging from 30 to 35 klx. The critical outdoor illuminance threshold for effective natural daylight is 4,500 lx. The region exhibits pronounced seasonal variations in daylight distribution. During summer, reduced cloud cover and higher solar altitude angles provide favorable outdoor daylighting conditions, particularly between 10:00 and 14:00, when sufficient direct sunlight typically satisfies daily daylighting requirements. In contrast, winter conditions are characterized by increased cloud cover and lower solar altitude angles, resulting in reduced direct solar radiation and diminished outdoor illuminance. These seasonal fluctuations are especially evident around midday in December and January. Overall, compared with other photometric climate zones, the Chang-Zhu-Tan region receives relatively limited direct sunlight and is characterized by moderate daylight availability.

**Table 1:** Daylight climate zones of China.

Daylighting climate zone	Province / Municipality	Annual average total outdoor illuminance (Eq, klx)
Ⅰ	Qinghai, Yunnan, Xizang, Xinjiang	E_q_≥45
Ⅱ	Yunnan, Inner Mongolia, etc.	40≤E_q_<45
Ⅲ	Shanxi, Guangdong, etc.	35≤E_q_<40
Ⅳ	Hunan, Shanghai, Shandong, Guangdong, etc.	30≤E_q_<35
Ⅴ	Guangxi, Sichuan, etc.	E_q_<30

Daylighting simulations were conducted on the Grasshopper platform using the Honeybee and Ladybug plugins, coupled with the Radiance simulation engine. Meteorological data were retrieved from the EnergyPlus Weather database provided by the U.S. Department of Energy for Zhuzhou. Based on the annual daylighting characteristics of the region, dynamic daylighting simulations were conducted for typical classroom spaces. The simulation period was defined from 08:00–17:00 on 21 June under an overcast sky condition, with electric lighting switched off and doors and windows fully open. The number of light reflections was set to six. The analysis reference plane was positioned 750 mm above the finished floor level in functional spaces, and a glare evaluation point was located at the center of Classrooms 3 and 4. Interior furnishings were excluded from the simulation, with only permanent architectural elements—ceilings, floors, and walls—considered. The reflectance values of interior surfaces and the performance parameters of side windows are summarized in Table 2.

**Table 2:** Selection of materials and reflectivity values for each component.

Site	Design value of reflection ratio	Window frame type	Glass type	Visible light transmittance	Glass reflection ratio
Ceiling	0.75	Single-layer aluminum window	Common glass	0.89	0.08
Ground	0.30				
Wall surface	0.60				
External surface	0.50				

### 2.2 Field measurement

#### 2.2.1 Determination of daylighting evaluation indicators.

The quality of natural daylighting in buildings is commonly evaluated using three static indicators: illuminance, daylight factor (DF), and daylight uniformity [22, 23]. According to the Chinese national standard GB 50033–2013 (Standard for Daylighting Design of Buildings), minimum daylighting requirements are specified on a standard working plane located 750 mm above the floor. For general classrooms with side daylighting, a daylight factor of no less than 3% and a minimum indoor natural illuminance of 450 lx are required. In addition, GB/T 50034–2024 (Standard for Lighting Design of Buildings) stipulates that the illuminance uniformity (U₀) on the desktop reference plane in educational spaces should not be less than 0.7. In practice, however, daylighting assessments are often based on instantaneous measurements or simulations conducted at specific time points. Although such results may satisfy standard requirements under those conditions, they do not adequately reflect the long-term daylighting performance of classroom spaces. Consequently, reliance on static indicators alone is insufficient for a comprehensive and objective evaluation of the indoor lighting environment in educational buildings.

The Chinese national standard GB/T 50378–2019 (Assessment Standard for Green Building) introduces time-dependent parameters into the evaluation of building performance. It requires that, in public buildings, at least 60% of the floor area in primary functional spaces achieve compliant natural illuminance levels for an average duration of no less than 4 hours per day.

Static indicators such as daylight factor, illuminance, and daylight uniformity are effective in describing instantaneous daylighting conditions. In contrast, dynamic indicators—including the compliance area ratio and compliance duration—capture daylighting performance over extended time periods and more accurately reflect actual indoor daylighting conditions. Accordingly, for educational buildings, the combined application of static and dynamic evaluation metrics, as summarized in Table 3, enables not only the assessment of compliance with daylighting standards but also a more comprehensive evaluation of the overall rationality and quality of the indoor lighting environment.

**Table 3:** Daylighting evaluation index.

Evaluating indicator	Standard value	Evaluation method
Coefficient of lighting	3%	Static evaluation
Illumination value	450 lx	Static evaluation
Uniformity of daylighting	0.7	Static evaluation
Hours of reaching the target for 60% of the area	4 h/d	Dynamic evaluation

#### 2.2.2 Typical Scene Screening.

This study takes the Chongye Building at Hunan University of Technology in Zhuzhou, Hunan Province, as the case study. Located on the university’s new campus, the Chongye Building is a six-story, I-shaped, interior corridor–type facility primarily used for teaching and research activities. The rectangular teaching units are arranged along a north–south axis, with classrooms relying on single-sided windows for daylight admission. Given that the first floor typically exhibits the most unfavorable daylighting conditions, two representative classrooms on this level were selected for detailed analysis: Classroom 3, a north-facing space with a room depth greater than 9000 mm, and Classroom 4, a north-facing space with a room depth of less than 9000 mm. Both classrooms have high occupancy and usage frequencies (Fig 2 and Table 4).

**Table 4:** Classroom Specifications.

Classroom scene	Depth(m)	Width(m)	Height(m)	External Corridor Type	Window-to-Floor Ratio
3#	13.5	8.8	3800	Interior corridor	0.171
4#	8.8	7.0	3800	Interior corridor	0.213

**Fig 2:**
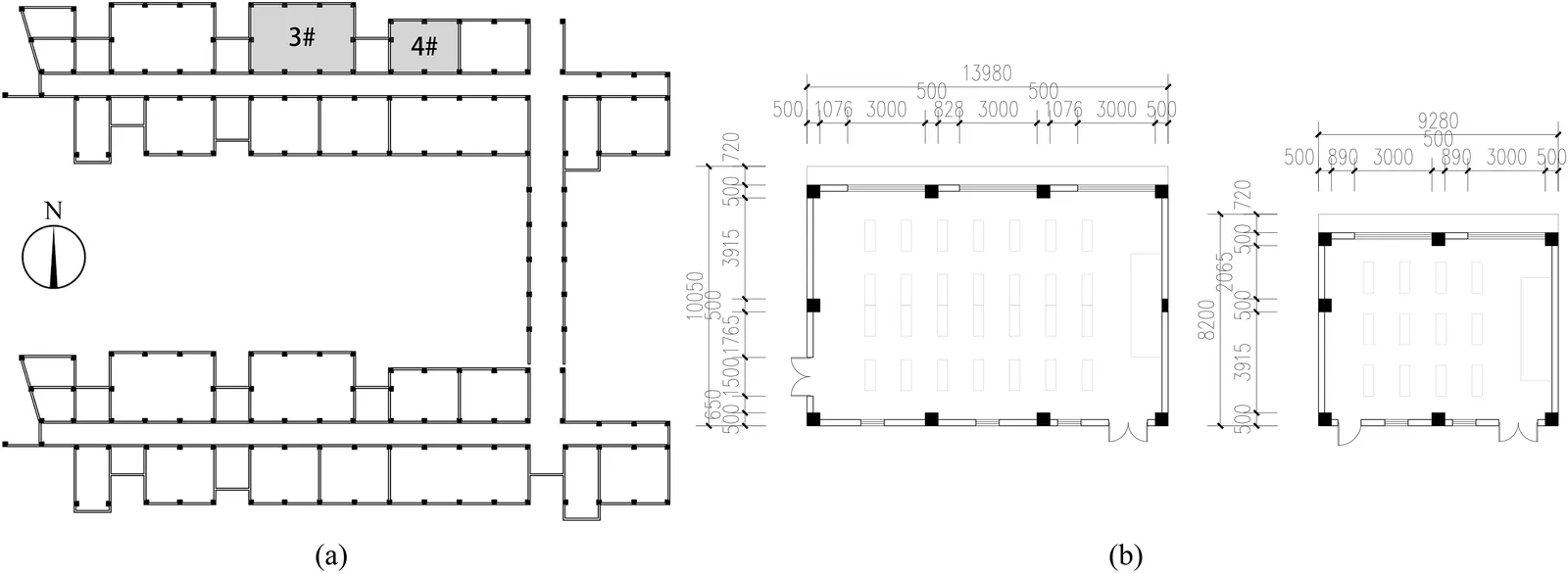
Screening of typical classroom scenarios in Chongye Building: (a) floor plan of the first floor of Chongye Building; (b) floor plans of typical classrooms 3# and 4#.

Daylighting performance tests were conducted in a representative classroom on 21 June, 24 June, and 3 July 2022 to measure actual indoor daylighting conditions. During the measurement periods, the outdoor sky was predominantly overcast, with total cloud cover approaching 80%. Outdoor horizontal illuminance ranged from 2,000–5,000 lx, closely approximating standard overcast sky conditions. Measurements were performed between 09:00 and 17:00, corresponding to peak teaching activity hours. During the tests, shading effects from surrounding trees and adjacent buildings were neglected. Electric lighting was switched off, and all curtains were fully opened to ensure unobstructed daylight admission.

#### 2.2.3 Instrument and equipment.

The measurement instruments utilized in this study include an illuminance meter, a luminance meter, a laser rangefinder, and a steel tape measure. Their specific models and applications are detailed in Table 5.

**Table 5:** Measurement Instruments and Associated Parameters.

Equipment and Models	Measurement Range	Application
Illuminance Meter/testo 540	0-19999lux	Measurement of illuminance
Luminance Meter/XYL-Ⅲ	0.1-100,000 cd/m^2^	Measurement of luminance
Laser Rangefinder/GLM 4000	40m	Measurement of distance
Steel Tape Measure/5m	5m	Measurement of distance

The natural light-guiding device adopted in this study is a non-tracking system based on the principle of specular reflection. The device, which has been granted a national utility model patent [21], consists of three main components: a light-concentrating unit, a light-guiding unit, and a diffuse reflection unit. It operates within the visible light spectrum (392–780 nm) and achieves a color rendering index of 100 (Fig 3).

**Fig 3:**
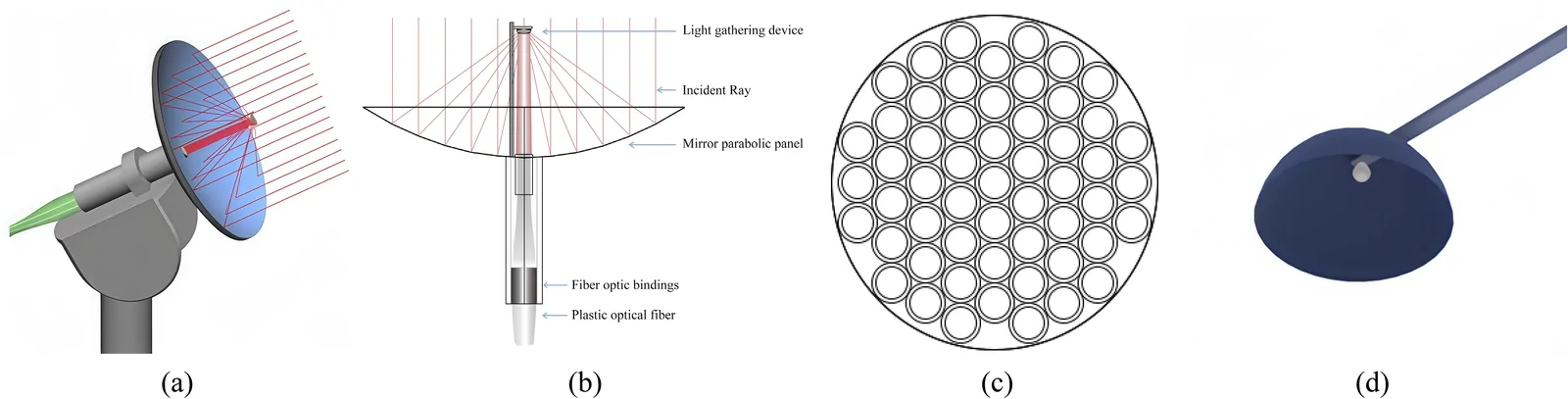
Schematic diagram of the natural light guiding device structure: (a) light-converging component; (b) mirror reflecting plate; (c) bundle of light conduction unit; (d) light-dispersing component.

The light-concentrating unit primarily comprises a parabolic reflector panel and a light-collection module, which concentrate incident daylight through reflective focusing. The parabolic reflector redirects outdoor natural light toward the receiving surface via optical focusing. The light-guiding unit employs plastic optical fibers as the transmission medium, each with a diameter of 2 mm. Owing to total internal reflection, these fibers are capable of transmitting high luminous flux, with a visible light transmittance of approximately 97% [4,24]. Each transmission module consists of 50 optical fibers connected to both the concentrating and diffusing units, producing a light beam with a diameter of approximately 80 mm (excluding fiber insulation).

The diffuse reflection unit adopts a lampshade-type structure to evenly disperse the transmitted daylight into the interior space. The light-diffusing aperture is arranged perpendicular to the normal direction at a height of 2500 mm above the finished floor level. Considering attenuation losses in plastic optical fibers, a single light-guiding device can supply 4–6 diffuse reflection apertures, thereby forming a complete indoor daylighting system.

The light-guiding device consists of three primary components: a concentrating unit, a transmission unit, and a diffusing unit. The concentrating unit comprises a curved mirror reflector and a lens. The curved mirror reflector has a diameter of 1000 mm and is directly connected to the transmission unit at its geometric center.

For vertical installation, the concentrating unit is mounted adjacent to the upper floor slab, with the curved mirror reflector fixed parallel to the slab. The optical fiber cables of the transmission unit are routed into individual rooms through pre-installed conduits integrated within the building’s electrical distribution system.

In terms of orientation, the concentrating unit is installed indoors, with the curved mirror reflector positioned on architectural protrusions such as window-mounted air-conditioning units or window sills. The installation angle is maintained between 30° and 45° to ensure effective daylight collection. The system configuration is not constrained by floor level, allowing flexible application across different stories of the building.

Compared with conventional light-guiding systems, the proposed natural light-guiding device demonstrates several advantages in architectural applications. First, it offers a relatively low cost. By eliminating complex components such as ray-tracing mechanisms and optical couplers, and instead employing cost-effective materials including plastic optical fibers and high-reflectivity coatings, the unit cost of the device is approximately 65 RMB per set, which is substantially lower than that of conventional commercial light-guiding systems. Second, the device features simple installation and operation, requiring only minimal modifications to existing walls and structural elements. Third, the system exhibits high cost-effectiveness, achieving efficient daylight conversion while maintaining low installation and operational complexity, thereby indicating strong potential for widespread implementation in educational buildings.

A single light-guiding device was installed in Classroom 4 and connected to one diffuse reflection outlet (Fig 4). The diffuse outlet was positioned at a height of 2500 mm above the finished floor level, while illuminance measurements were conducted on a horizontal plane at 750 mm above the floor. With the diffuse outlet activated and all doors, windows, and curtains closed, illuminance data were recorded at hourly intervals, yielding nine measurement points per day. These measurements were used to assess the actual illuminance contribution of the light-guiding device and to determine its effective light-guiding radius within the classroom space.

**Fig 4:**
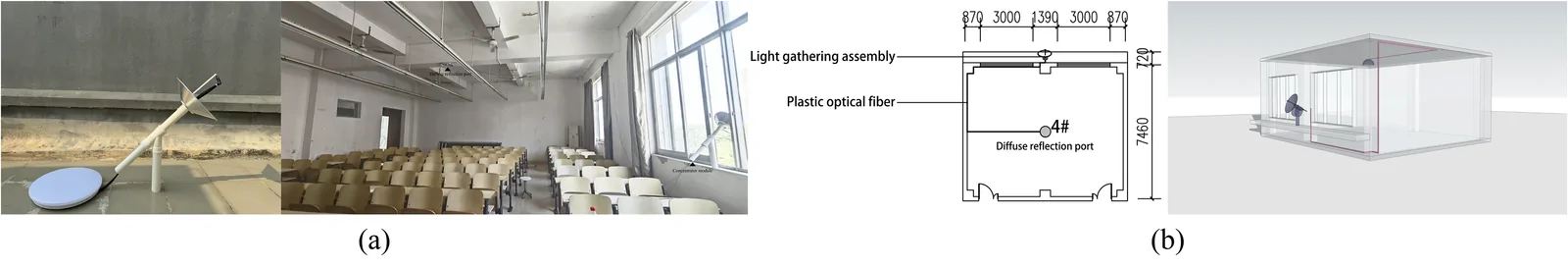
Efficacy test: (a) efficacy test field; (b) model of efficacy test.

### 2.3 Measured results

Illuminance measurement points were arranged at 2000 mm intervals along both the width and depth directions of Classrooms 3 and 4, with all points located on a horizontal reference plane 750 mm above the finished floor level. Measurements were conducted at three representative zones within each classroom: the window-adjacent area, the central area, and the area farthest from the windows (Figs 5 and 6).

**Fig 5:**
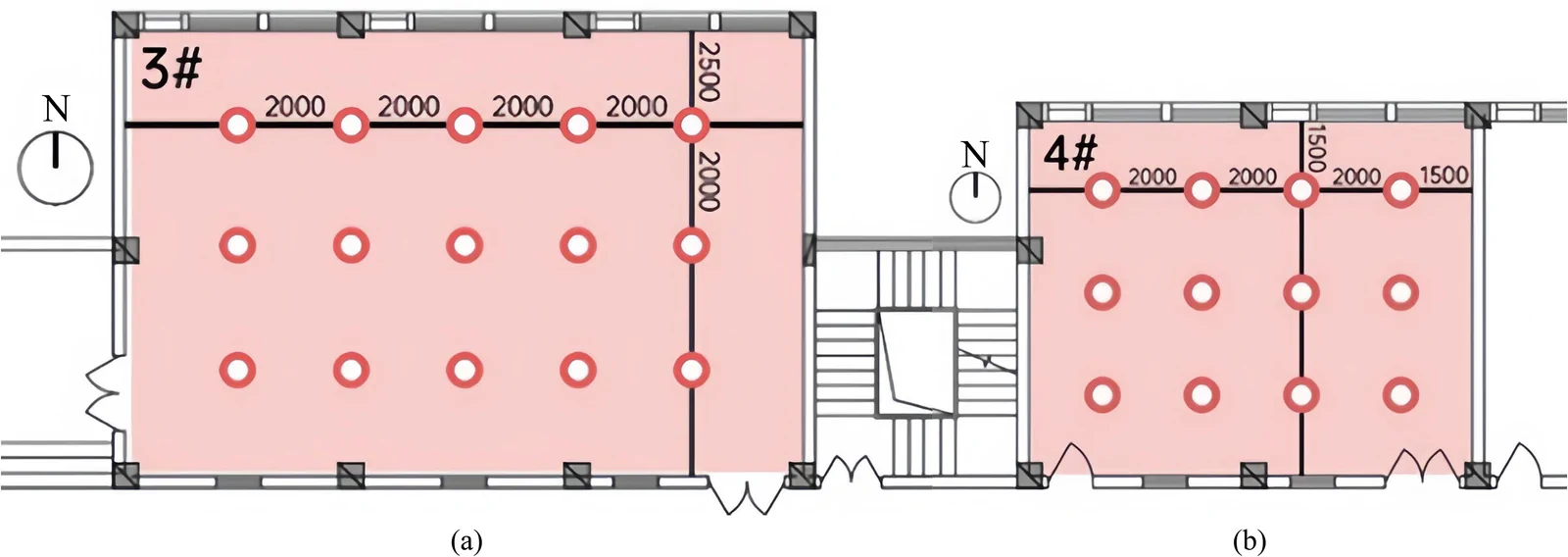
Distribution of measurement points in the classroom: (a) 3#; (b) 4#.

**Fig 6:**
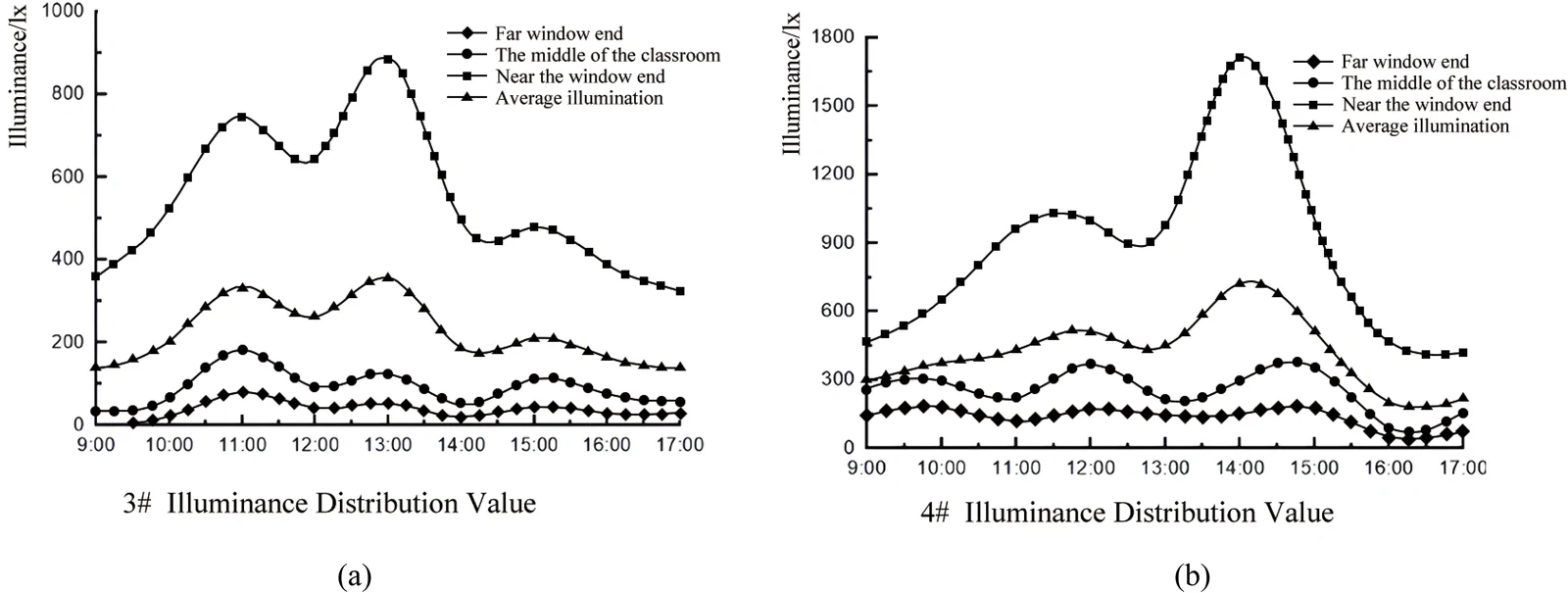
Average illuminance distribution of a typical classroom: (a) 3#; (b) 4#.

Table 6 indicates that the overall lighting performance on the first floor of the teaching building is inadequate. The average daylight factor is below 3%, and the mean indoor illuminance is less than 300 lx, both of which fail to meet recommended standards for teaching spaces. The effective daylight penetration depth is approximately 4000 mm in north-facing classrooms and about 4300 mm in south-facing classrooms. Pronounced glare is observed in window-adjacent zones on both orientations. Daylighting in the interior corridor relies primarily on exterior windows at both ends of the corridor, with additional contribution from adjacent outdoor lounge spaces. However, the average daylight factor in the corridor is only 1%, and although localized areas meet basic usage requirements, illuminance decreases substantially toward the central zone.

**Table 6:** Comparison of Measured and Simulated Illuminance.

Classroom scene	Maximum illuminance (lx)		Minimum illumination (lx)		Average illumination (lx)	
	Actual measurement	Simulation	Actual measurement	Simulation	Actual measurement	Simulation
3#	888	915	12	13	220	235
4#	1712	1790	53	56	412	433

In Classroom 3, illuminance levels near the window were relatively high, with an average value of 556 lx and a maximum of 888 lx recorded at 13:00. In contrast, illuminance at the zone farthest from the window exhibited severe attenuation, with a peak value of only 12 lx and an average of 36 lx. This pronounced spatial gradient resulted in poor daylight uniformity across the classroom. Overall, the mean illuminance in Classroom 3 was approximately 220 lx, indicating insufficient natural daylighting and failing to satisfy the illuminance requirements for routine teaching activities.

At the window-adjacent zone of Classroom 4, illuminance reached a peak value of 1,712 lx at 14:00, while the minimum value of 53 lx occurred at 17:00. The zone farthest from the window exhibited peak illuminance levels of 169 lx and 171 lx during the periods of 09:30–10:00 and 14:40–15:00, respectively. At 14:00, illuminance decreased markedly with increasing distance from the window, indicating limited daylight penetration. The average indoor illuminance in Classroom 4 was approximately 400 lx, with values exceeding 450 lx only between 12:00 and 15:00. Similar to Classroom 3, daylight distribution was highly non-uniform, increasing the potential risk of visual discomfort and glare.

Overall, despite favorable outdoor daylight conditions and the absence of artificial lighting, multiple areas in both classrooms failed to meet the prescribed illuminance standards (Fig 6).

### 2.4 Simulation results

An average outdoor illuminance of 3,000 lx, measured during the monitoring period, was used as the input condition for daylighting simulations of Classrooms 3 and 4 conducted on the Grasshopper platform (Fig 7). Using the measured outdoor horizontal illuminance during the monitoring period as the input boundary condition for the simulation model, the simulated maximum, minimum, and average illuminance levels in the classrooms were extracted and compared against the corresponding empirical data under identical operating conditions. The relative error and Mean Absolute Percentage Error (MAPE) were subsequently employed to validate the accuracy and reliability of the model.

**Fig 7:**
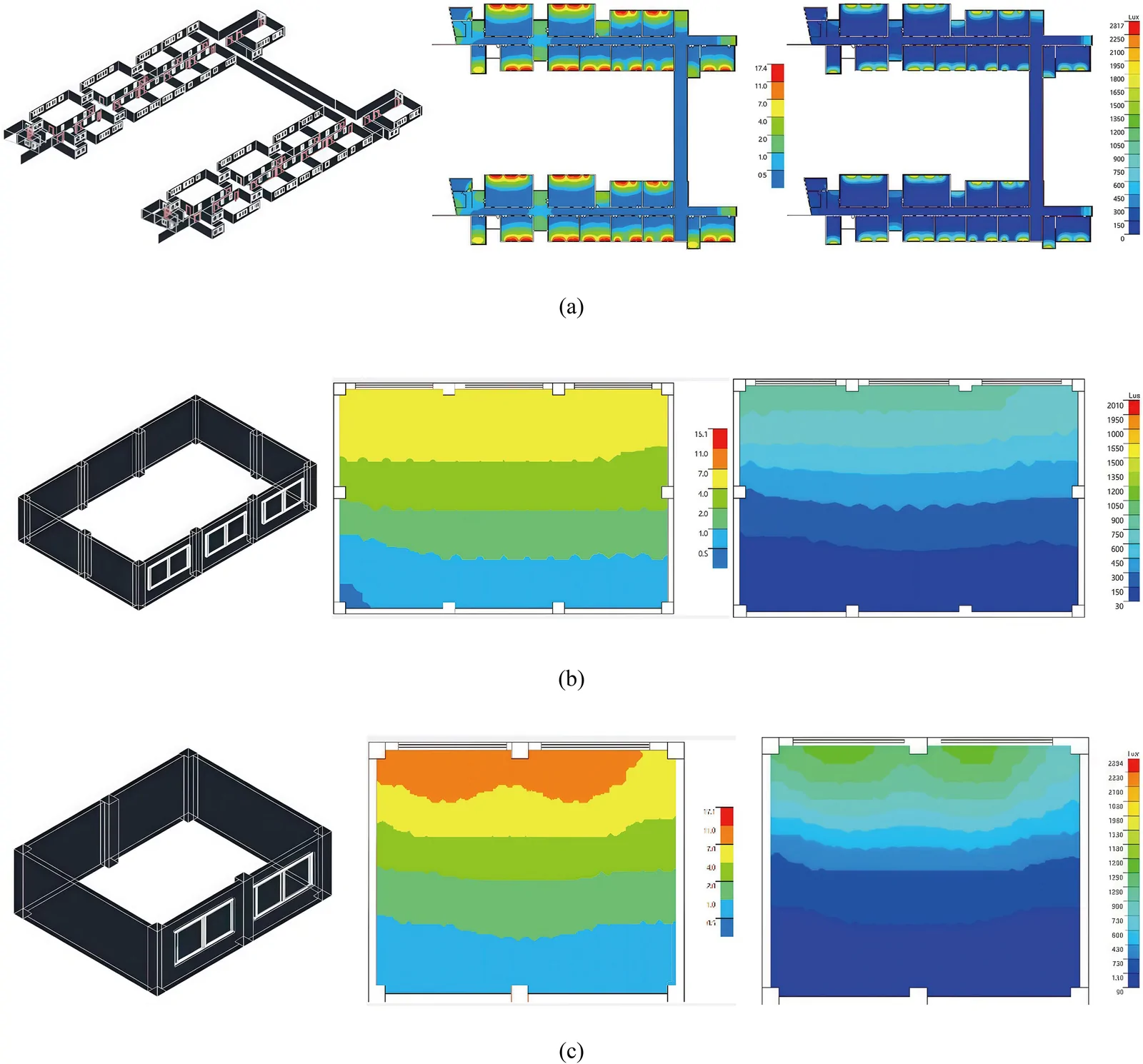
Plane, daylight factor cloud diagram, and illuminance value cloud diagram: a: The first floor of Chongye Building. b: 3#. c: 4#.

The relative error for an individual indicator was calculated using the following equation:


$$\begin{array}{c} \delta_{i}=\frac{\left|E_{sim,i}-E_{mea,i}\right|}{E_{mea,i}}\times \text{1}\text{0}\text{0}\% \end{array}$$ (1)

where:


$$\begin{array}{c} \delta_{i}-the\;relative\;error\;of\;the\;i-th\;indicator\;(\%) \end{array}$$

$$\begin{array}{c} E_{sim,i}-simulated\;statistical\;illuminance\;value\;(lx) \end{array}$$

$$\begin{array}{c} E_{mea,i}-measured\;statistical\;illuminance\;value\;(lx) \end{array}$$

The Mean Absolute Percentage Error (MAPE) was calculated using the following equation:


$$\begin{array}{c} MAPE=\frac{\text{1}}{k}\sum_{i=\text{1}}^{i}\frac{\left|E_{sim,i}-E_{mea,i}\right|}{E_{mea,i}}\times \text{1}\text{0}\text{0}\% \end{array}$$ (2)

where:

k *—the number of statistical indicators included in the validation*

Substituting the empirical data from Table 6 into the equations, the calculated results are summarized in Table 7:

**Table 7:** Daylighting simulation model reliability and error evaluation.

Classroom scene	Statistical Metric	Measured Value/lx	Simulated Value/lx	Relative Error/%	MAPE/%
3#	Maximum Illuminance	888	915	3.04	5.59
	Minimum Illuminance	12	13	8.33	
	Average Illuminance	220	235	6.82	
4#	Maximum Illuminance	1712	1790	4.56	
	Minimum Illuminance	53	56	5.66	
	Average Illuminance	412	433	5.1	

As indicated by the calculated results, the relative errors for all statistical metrics are predominantly around 5%, and the overall MAPE falls well within the acceptable threshold for daylighting simulations. This demonstrates strong agreement between the simulated outputs and empirical measurements, confirming that the established luminous environment model exhibits high fidelity and reliability for subsequent simulation analyses of hybrid daylighting patterns.

As summarized in Table 8, Classrooms 3 and 4 exhibit average illuminance values of 355 lx and 383 lx, respectively, with corresponding average daylight factors of 2.64% and 2.84%. The mean daylight uniformity ratios are 0.18 for Classroom 3 and 0.17 for Classroom 4, indicating uneven daylight distribution. In both classrooms, illuminance levels decrease markedly toward the zones farthest from the windows, resulting in multiple areas with insufficient lighting. Conversely, window-adjacent areas display relatively high illuminance and daylight factor values, accompanied by evident glare. Moving deeper into the classrooms, glare intensity gradually diminishes, with daylight glare index values of 11.7 for Classroom 3 and 10.5 for Classroom 4.

**Table 8:** Light environment index of typical classrooms in Chongye Building.

Typical classroom	Orientation	Window-floor ratio	Average illumination (lx)	Average daylight factor (%)	Average daylighting uniformity	Standard area ratio + Standard duration (h/d)	Glare index
3#	North	11%	355	2.64	0.18	61% + 7.9	11.7
4#	North	15%	383	2.84	0.17	45% + 7.5	10.5

### 2.5. Summary of measured and simulated results

Based on the combined results of on-site measurements and daylighting simulations for the two representative classrooms, several deficiencies in the lighting environment of the Chongye Building were identified:

Insufficient indoor daylight availability. Both classrooms rely exclusively on side-window daylighting and fail to meet the prescribed design requirements for illuminance (450 lx) and daylight factor (3%). The limited daylight penetration depth results in extensive underlit zones along the classroom length, extending up to approximately 4500 mm in Classroom 3 and 3500 mm in Classroom 4, corresponding to nearly half of the total room depth in each case.

Inadequate daylighting quality. Beyond non-compliant areas, pronounced glare occurs in window-adjacent zones, and overall daylight uniformity is poor. Significant variations in illuminance and daylight factor are observed between the two classrooms. In Classroom 3, areas meeting the 450 lx illuminance criterion account for approximately 61% of the total floor area, with an average daily compliance duration of 7.9 h. In contrast, in Classroom 4, compliant areas constitute less than 60% of the interior space.

These deficiencies are representative of corridor-style teaching buildings across universities in the Chang-Zhu-Tan region. Daylighting patterns that rely solely on side windows are insufficient to support routine teaching activities, while increased dependence on electric lighting leads to higher building energy consumption. The adoption of hybrid daylighting systems that integrate natural daylight with light-guiding devices offers a viable design strategy to optimize classroom lighting environments without additional energy input, thereby addressing both visual comfort and energy efficiency concerns.

### 2.6 Arrangement of natural light guide devices

Dynamic daylighting simulations were conducted to determine the effective depth of natural daylight penetration in classroom areas that satisfy the established lighting evaluation criteria, thereby identifying zones requiring supplementary illumination. Based on the depth and width of the classrooms, natural light-guiding devices were then strategically arranged to enhance lighting performance within these underlit zones. This process enables the development of a performance-based hybrid daylighting system that integrates natural daylight with artificial light-guiding technology.

The analysis indicates that the overall lighting conditions in both classrooms are inadequate. In window-adjacent zones, the average illuminance reaches approximately 450 lx, with an average daylight factor of 3%. However, both illuminance and daylight factor decrease rapidly with increasing distance from the windows. In Classroom 3, approximately 61% of the floor area achieves the 450 lx criterion, corresponding to an effective daylight penetration depth of 4500 mm. In Classroom 4, only about 45% of the area meets the same illuminance requirement. Consequently, supplementary daylighting devices are required for the remaining 39% of Classroom 3 and 55% of Classroom 4.

The four hybrid daylighting patterns (HDL, MDL, TL, and LDL) were generated via rule-based spatial layouts rather than algorithmic global optimization. First, a 2000 mm spacing between adjacent devices was established based on classroom geometric dimensions and the effective daylighting radius of the system (Fig 8). This arrangement ensures that the supplementary illumination accurately covers deep-plan daylight-deficient zones while avoiding glare-sensitive areas. Second, these four patterns are designed to strategically deploy devices within the daylight-deficient areas along the room depth, achieving differentiated luminous environmental quality through variations between horizontal illuminance uniformity and the number of installed devices.

**Fig 8:**
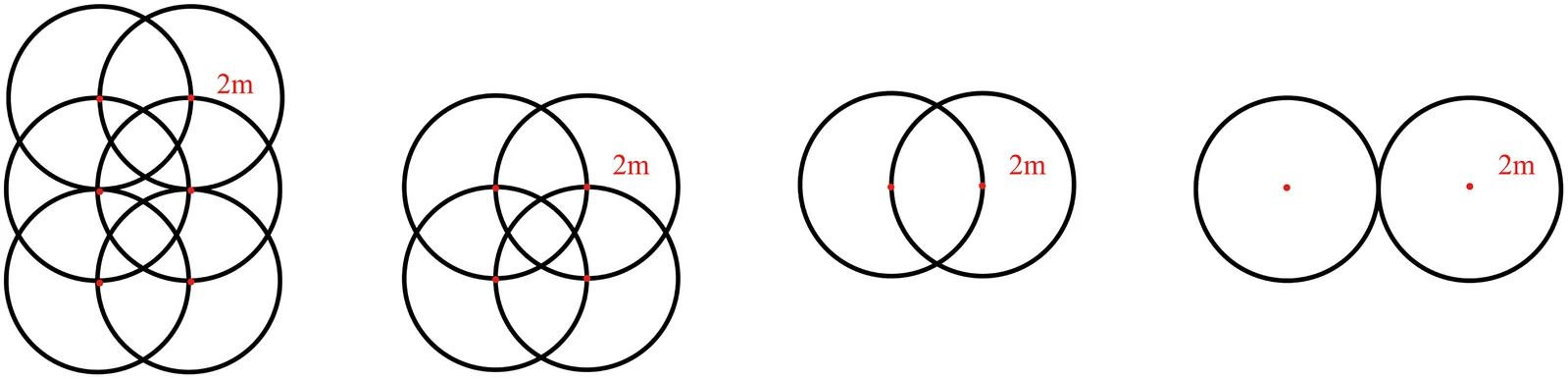
Schematic Diagram of Device Layout.

Based on the effective lighting radius of the light-guiding devices and the calculated daylight penetration depths, four hybrid daylighting patterns were developed: High-Density Layout (HDL), Medium-Density Layout (MDL), Targeted Layout (TL), and Low-Density Layout (LDL). Daylighting performance contour maps for relevant indicators were generated through simulation (Fig 9).

**Fig 9:**
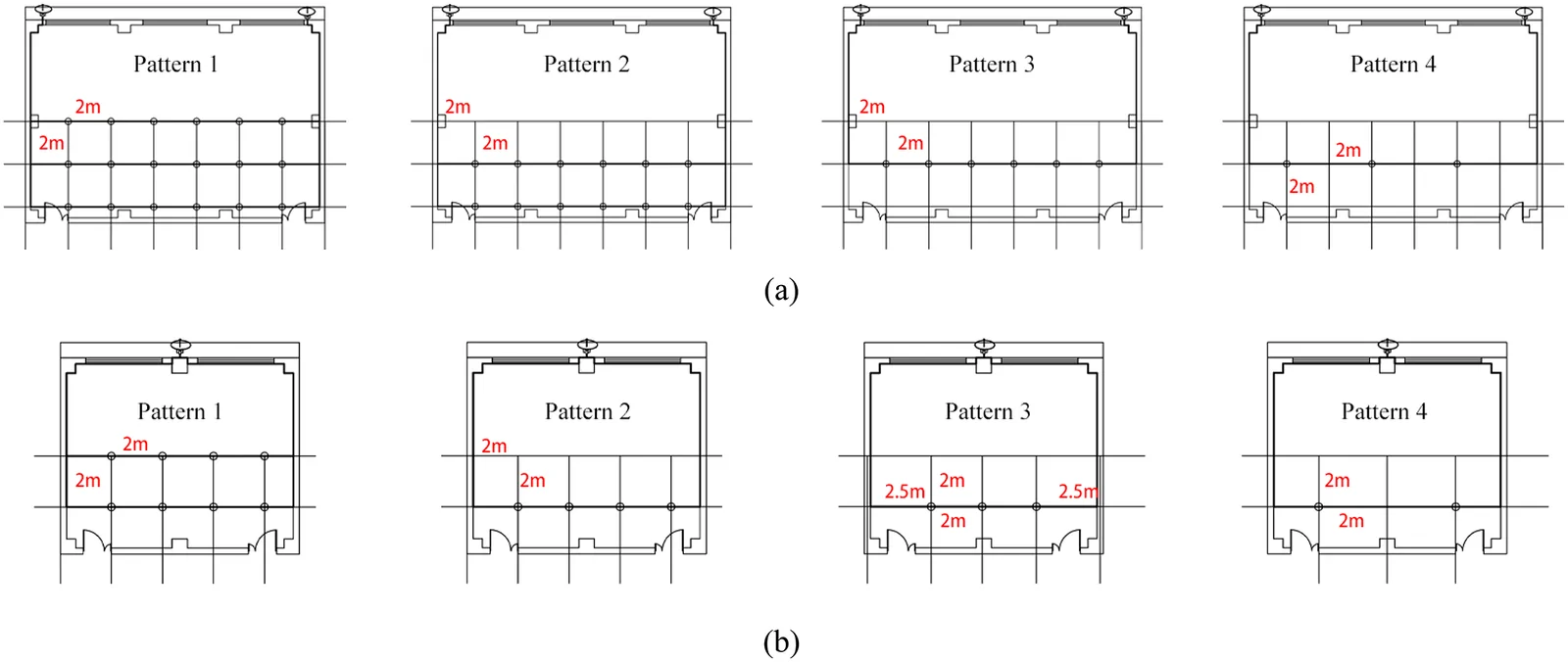
Four kinds of mixed daylighting patterns: (a) 3#; (b) 4#.

Pattern 1: High-Density Layout (HDL)

In this pattern, the installation of light-guiding devices begins at the boundary of the supplementary lighting zone, with devices arranged at 2000 mm intervals. The layout strictly follows the experimentally determined daylighting deficiencies, aiming to maximize illuminance levels across the entire interior area.

Pattern 2: Medium-Density Layout (MDL)

In this mode, installation starts 2000 mm from the boundary of the supplementary lighting zone, with devices spaced at 2000 mm intervals. Considering the effective daylight penetration depth associated with single-sided window daylighting, the system is offset toward the interior to primarily compensate for insufficient daylight in zones farthest from the windows.

Pattern 3: Targeted Layout (TL)

For Classroom 3, device installation begins 2000 mm from the boundary of the supplementary lighting zone, with devices arranged at 2000 mm intervals. For Classroom 4, installation also starts 2000 mm from the boundary; however, the devices are configured in centrally located groups of three. This layout aims to ensure overall compliance with lighting standards while placing particular emphasis on improving spatial daylight uniformity.

Pattern 4: Low-Density Layout (LDL)

In this pattern, installation in Classroom 3 begins 2000 mm from the boundary of the supplementary lighting zone, with devices spaced at 4000 mm intervals. In Classroom 4, installation also starts 2000 mm from the boundary, with devices arranged in centrally located pairs. The objective of this layout is to reduce the number of devices while maintaining basic indoor lighting requirements and supporting routine teaching activities.

### 2.7 Energy-efficient design

According to GB 50033–2013, the annual lighting electricity savings contributed by natural daylighting should be calculated using the following formula:


$$\begin{array}{c} W_{e}=\sum (P_{n}\times t_{D}\times F_{D}+P_{n}\times t_{D}^{\prime }\times F_{D}^{\prime })/\text{1}\text{0}\text{0}\text{0} \end{array}$$ (3)

where:


$$\begin{array}{c} W_{e}-Annual\;lighting\;electricity\;savings\;(kWh/a) \end{array}$$

$P_{n}-Total\;installed\;lighting\;power\;of\;the\;room\;or\;zone\;(W)$

$t_{D}-$ *Hours of full utilization of natural daylighting (h), taken as 1619 for educational buildings*


$$\begin{array}{c} F_{D}-Daylighting\;dependence\;coefficient\;during\;full\;utilization\;of\;natural \end{array}$$

$$\begin{array}{c} daylighting,\;taken\;as\;\text{1} \end{array}$$

$t_{D}^{\prime }-$ *Hours of partial utilization of natural daylighting (h), taken as 429 for educational buildings*


$$\begin{array}{c} F_{D}^{\prime }-Daylighting\;dependence\;coefficient\;during\;partial\;utilization\;of\;natural \end{array}$$

$$\begin{array}{c} daylighting,\;taken\;as\;\text{0}.\text{5} \end{array}$$

Taking Hunan University of Technology as a case study, the electricity price is set at 0.6 RMB/(kW·h). Consequently, the annual monetary savings on electricity can be calculated by multiplying the annual lighting electricity savings by the unit electricity price.

## 3. Results

As summarized in Table 9, the natural light-guiding device exhibits an average outdoor-to-indoor light conversion efficiency of 42.08%, where the conversion efficiency is defined as the ratio of the indoor output illuminance of the device (β) to the incident outdoor sky illuminance (α). Simultaneously, dynamic daylight-guidance simulations conducted on the Grasshopper platform under standard overcast skies indicated that the area directly beneath the diffuse reflection port reached a maximum average illuminance of 555 lx. The illuminated profile formed a concentric circle with an effective daylighting radius of approximately 1500 mm, demarcated by the threshold of ≥450 lx from the central aperture to the periphery.

**Table 9:** Statistical data of the device daylighting efficacy test.

All overcast day	Testing time (AM-PM)	Average outdoor illuminance value (lx)	Average outdoor sky incident illuminance value α (lx)	The output illuminance value β of the diffuse reflection port (lx)	Average single group conversion rate (%)	Average conversion rate (%)
9	2 133	855	357	41.75	42.08
10	2 569	990	458	46.26	
11	3 900	1 350	511	37.85	
12	4 511	1 650	601	36.42	
13	4 597	1 782	714	40.06	
14	4 610	1 799	764	42.46	
15	4 813	1 811	649	38.53	
16	3 121	1 005	500	49.75	
17	2 511	987	451	45.69	

Daylighting simulations for the four proposed hybrid layout patterns were subsequently performed using the Grasshopper platform (Figs 10 and 11).

**Fig 10:**
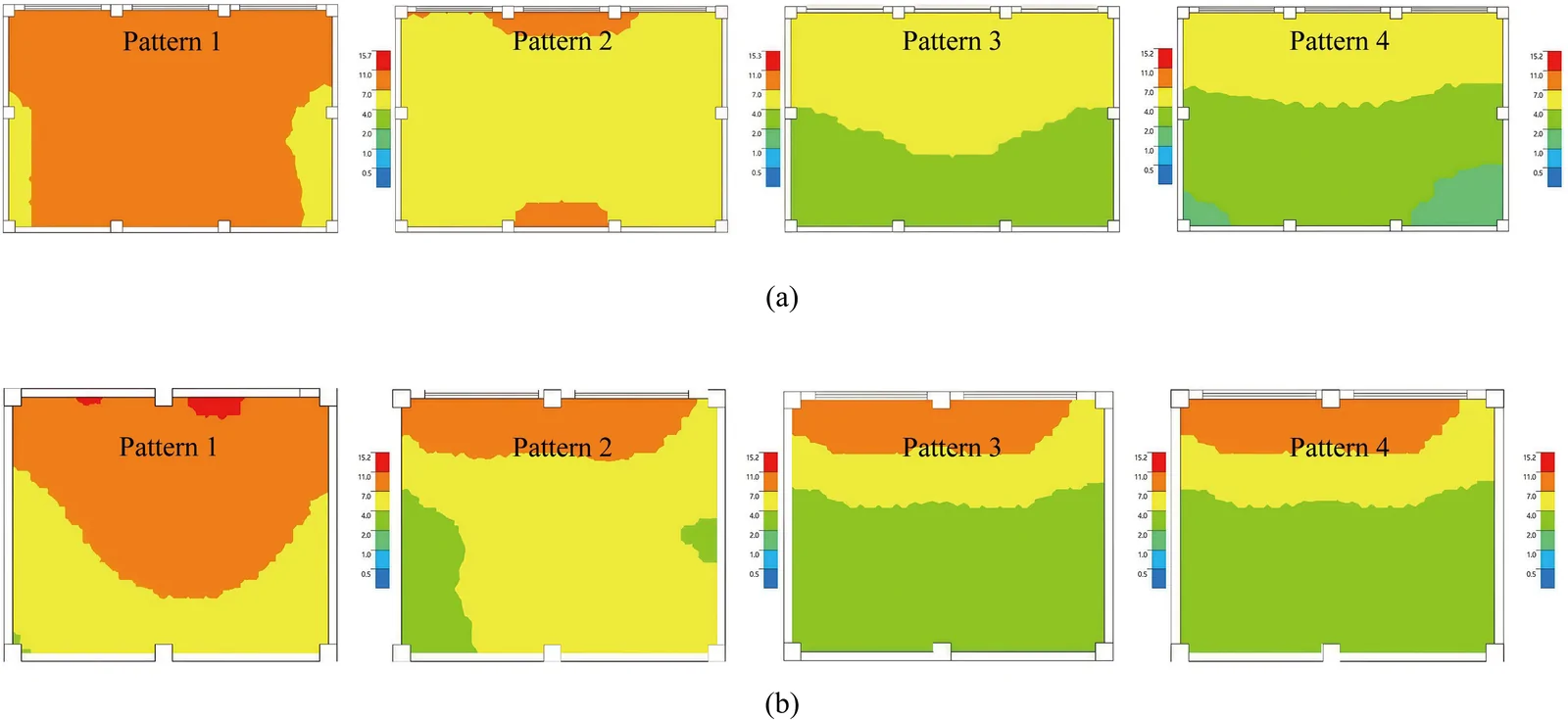
Cloud diagram of daylighting coefficient in classrooms under four hybrid daylighting patterns: (a) 3#; (b) 4#.

**Fig 11:**
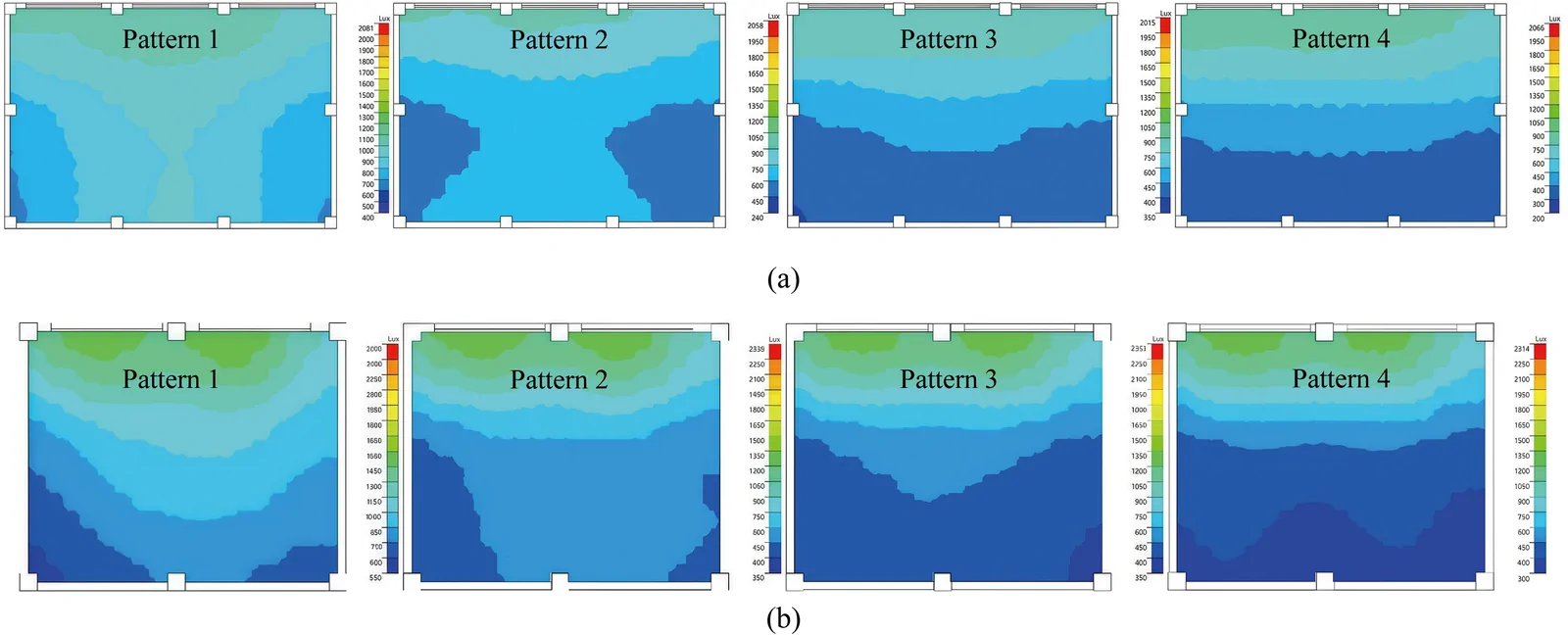
Classroom illuminance cloud diagram under the four hybrid lighting patterns: (a) 3#; (b) 4#.

(1) HDL employs the highest number of natural light-guiding devices and demonstrates superior performance across all evaluated indicators. It achieves the most favorable lighting conditions throughout the classroom while effectively reducing glare compared with scenarios without light-guiding devices.
(2) MDL prioritizes improvements in daylighting performance in zones distant from the windows by reducing device density near the window area. Despite this adjustment, all performance indicators remain well above the prescribed standards, ensuring adequate and stable indoor illumination.
(3) TL presents localized areas with insufficient illuminance. The proportions of classroom area meeting the illuminance criteria reach 99%, 97%, and 94%, respectively. However, the glare index decreases continuously.
(4) LDL employs the fewest natural light-guiding devices and exhibits the lowest performance across all daylighting metrics compared to the preceding three patterns. In particular, the daylighting performance near the side wall at the far-window end is relatively weak; however, it still satisfies the evaluation standards while offering the optimal economic efficiency.

Based on a comprehensive evaluation using four indicators—daylight factor, illuminance, daylight uniformity, and the ratio and duration of effectively daylit area—the four hybrid daylighting modes achieved static and dynamic daylighting performance that met and exceeded the relevant standards. Moreover, the effective daylight penetration depth reached its maximum under all four modes (Table 10 and Fig 12).

**Table 10:** Statistics of Light Environment Indexes under Four Patterns.

Classroom	Pattern	Equipment	Average daylight factor (%)	Average illumination (lx)	Average daylighting uniformity	Standard area ratio + Standard time (h/d)	Glare index
3#	1	18	7.25	866	0.789	100% + 9.1	9.3
	2	12	5.90	684	0.754	100% + 8.9	9.9
	3	6	4.52	535	0.736	99% + 8.5	8.6
	4	3	3.54	454	0.718	82% + 8.1	8.1
4#	1	8	7.25	819	0.729	100% + 8.8	7.1
	2	4	4.98	605	0.712	100% + 8.5	7.3
	3	3	4.51	558	0.704	97% + 8.3	7.8
	4	2	3.95	493	0.702	85% + 8.1	6.8

**Fig 12:**
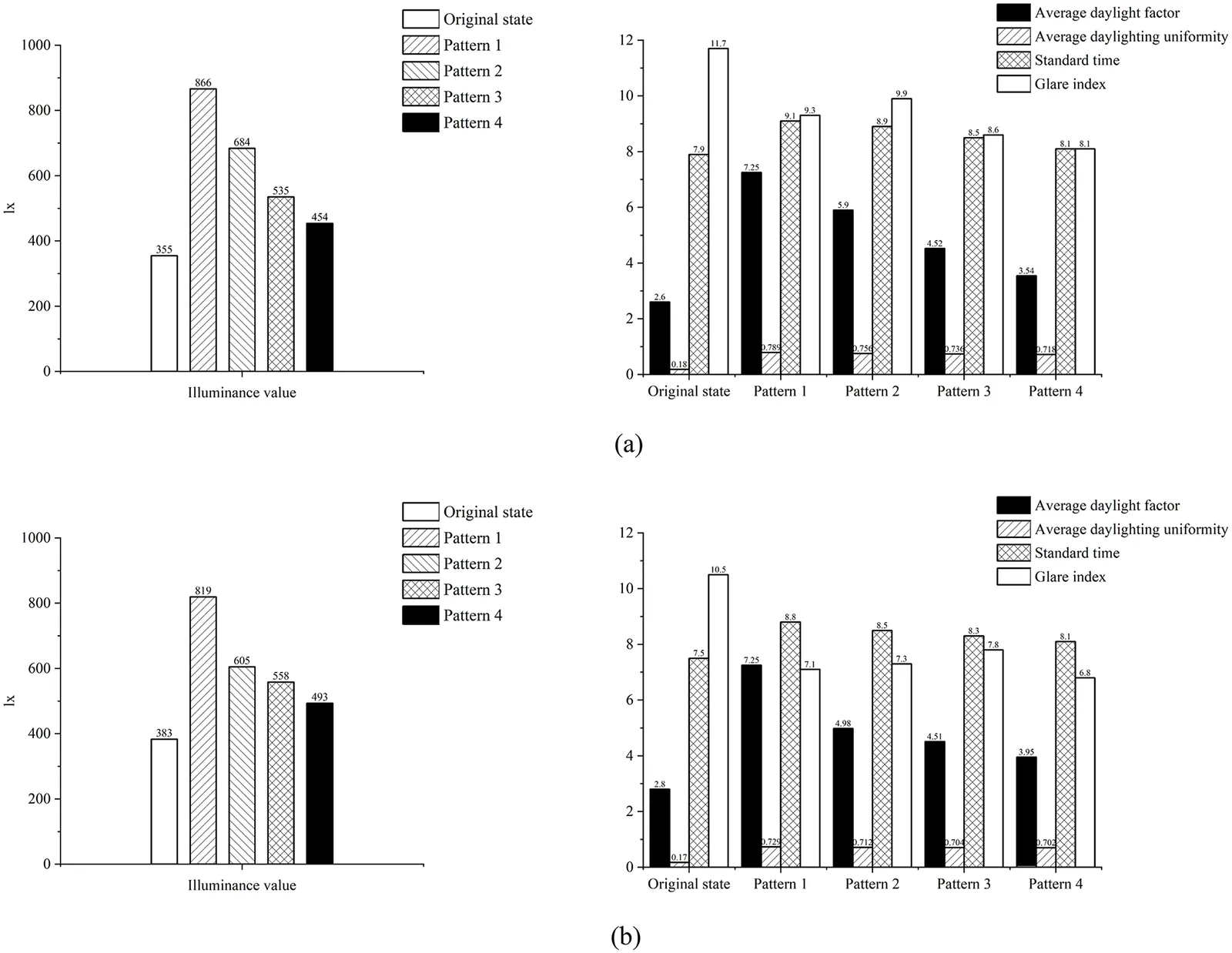
Indices of the classroom in four different patterns: (a) 3#; (b) 4#.

The four hybrid daylighting modes proposed in this study all achieve full utilization of natural daylighting. Implementing these modes can significantly reduce both lighting electricity consumption and power costs, with the specific calculation results summarized in Table 11.

**Table 11:** Statistics on annual electricity savings and electricity cost savings.

Classroom	Single-lamp power (W)	Number of lighting fixtures per classroom	P_n_ (W)	t_D_ (h)	F_D_	W_e_ (KWh/a)	W_e_ (J)	Annual electricity cost savings (RMB)
3#	40	27	1080	1619	1	1748.52	6.295 × 10^9^	1049.1
4#	40	12	480	1619	1	777.12	2.798 × 10^9^	466.3

The simulated reduction in annual electricity dependence confirms that retrofitting institutional space via dynamic supplemental deployment is an exceptionally viable path. Similar localized baseline optimization strategies in green building operations have demonstrated that targeting specific load predictions can systematically prevent fractional energy redundancy and secure high-efficiency performance boundaries [25].

## 4. Discussion

According to the field survey, large standard classrooms accommodating 60–80 students in corridor-style teaching buildings across the Chang-Zhu-Tan region typically exhibit depths ranging from 8200 to 12000 mm and widths from 9000 to 13000 mm. Small standard classrooms designed for 20–40 students generally have depths of 6200–8200 mm and widths of 7200–9200 mm. Since the typical classrooms selected in this study are comparable in scale to the interior-corridor classrooms in the Chang–Zhu–Tan region, the findings can provide guidance and references for the luminous environment optimization of interior-corridor classrooms sharing similar architectural characteristics and daylight climate conditions. There are two recommendations for future research:

(1) HDL and MDL are well-suited to larger classrooms with high lighting demands, such as drafting studios, laboratories, and art classrooms. By employing a higher density of light-guiding devices, these patterns achieve elevated illuminance levels across the interior space. MDL, in particular, reduces the number of fixtures near the window zone while providing targeted supplementary lighting in areas distant from the windows. For classrooms with greater depth or width, the layout can be extended in both directions based on a device spacing corresponding to the core effective radius of 1500 mm (or 2000 mm spacing for adjacent overlapping fields).
(2) TL and LDL are more appropriate for standard teaching classrooms. These patterns reduce the overall number of fixtures while emphasizing integration with side-window daylighting, thereby meeting illuminance requirements and significantly improving daylighting uniformity. LDL further accounts for operational and economic efficiency. In this hybrid lighting pattern, each fixture achieves an effective light-guiding radius of approximately 2000 mm, adequately satisfying the lighting environment requirements of both classroom types. The layout may be further expanded in depth and width directions using a 2000 mm effective radius per fixture.

The hybrid daylighting modes proposed in this study demonstrate significant lighting energy-saving potential in campus classroom scenarios. The results indicate that the data for Classrooms 3# and 4# exhibit an energy-saving pattern highly and positively correlated with the total installed lighting power, verifying that natural daylighting holds higher application value in spaces with elevated lighting loads.

From an economic perspective, both classroom scenarios exhibit stable cost-effectiveness. Based on an electricity tariff of 0.6 RMB/kWh, the annual electricity cost savings for a single classroom can reach the order of thousands of RMB, which can substantially lower long-term operational economic costs. This outcome further proves that the hybrid daylighting mode is highly generalizable across classrooms with diverse lighting patterns. The linear relationship observed between energy-saving performance and lighting load provides a robust quantitative basis for the daylighting and energy-saving retrofitting of campus buildings.

## 5. Conclusions

Taking the Chongye Building at Hunan University of Technology as a case study, this study investigates hybrid daylighting patterns integrating natural daylighting and artificial light-guiding, leading to the following conclusions:

First, based on the calculated effective daylight penetration depths for classrooms of different sizes, four hybrid daylighting patterns were proposed through the strategic arrangement of light-guiding devices. These patterns can be flexibly adapted according to classroom dimensions and functional requirements. HDL and MDL are recommended for larger classrooms with higher illuminance demands, while TL and LDL are more suitable for standard classrooms.

Second, the implementation of the proposed hybrid daylighting system led to substantial improvements in the lighting performance of interior corridor-style teaching buildings. Average illuminance, daylight factor, and daylight uniformity increased significantly, while the effective illuminated area expanded considerably. At the same time, the glare index was consistently reduced, resulting in a marked enhancement of the overall classroom lighting environment.

Finally, to achieve a comprehensive assessment of daylighting performance, four lighting evaluation indicators were established, including daylight factor, illuminance, daylight uniformity, and the combined compliance ratio of effective daylighting area and compliance duration. This integrated evaluation framework more accurately reflects the dynamic characteristics of classroom daylighting, providing a more scientific basis for assessing indoor lighting environments in educational buildings.

Despite the insights gained from this study, several limitations must be acknowledged. First, constraints exist regarding the research samples and simulation scenarios; this study relied on a single case study of the Chongye Building at Hunan University of Technology, resulting in relatively narrow coverage. Concurrently, the daylighting simulation was restricted to typical fully overcast sky conditions during the summer, without considering the dynamic, year-round variations in seasons, weather, and solar altitude angles, which introduce potential simulation tolerances. Second, the analytical dimensions of this study primarily focus on the physical indicators of the light environment, without incorporating physiological and psychological user responses, such as visual comfort, perception, and learning efficiency, into the evaluation framework. Furthermore, the hybrid daylighting modes were constructed solely based on classroom width and depth, omitting multi-dimensional variables such as classroom height, spatial morphology, shading facilities, and occupant activities. Lastly, the technical investigation of the apparatus and system-level analysis requires further deepening; the tubular daylighting device is still in the experimental stage, lacking long-term operational field data in actual educational scenarios, and coupling research involving intelligent management systems and optimization algorithms has yet to be conducted.

To overcome these limitations, future research can be advanced in the following directions. First, the scope of research samples should be expanded to encompass educational buildings across different climate zones and architectural typologies, while integrating year-round, multi-scenario simulations to calibrate model errors and enhance the generalizability and precision of the findings. Second, a comprehensive evaluation framework combining both physical indicators and physiological responses will be constructed, followed by multi-objective daylighting optimization research. Finally, long-term empirical field tests of the equipment should be carried out to establish a full-lifecycle economic benefit analysis model. By combining this with smart control systems for coupled research, an intelligent daylighting and energy-saving framework can be developed to facilitate the practical deployment and promotion of this technology.

## Supporting information

S1 File Measured indoor illuminance data of Chongye Building 3#. (Optional brief legend if needed, e.g., Raw indoor illuminance measurements collected during the monitoring period for classroom 3#).
(XLSX)

S2 File Measured indoor illuminance data of Chongye Building 4#. (Optional brief legend if needed, e.g., Raw indoor illuminance measurements collected during the monitoring period for classroom 4#).
(XLSX)
